# Malignant renal epithelioid angiomyolipoma with TFE3 gene amplification mimicking renal carcinoma 

**DOI:** 10.5414/CNCS109443

**Published:** 2018-05-24

**Authors:** Haili Wang, Haiyu Zhan, Zhigang Yao, Qingwei Liu

**Affiliations:** 1 Department of Radiology, and; 2 Department of Pathology, Provincial Hospital, Affiliated to Shandong University, Shandong Province, China

**Keywords:** malignant tumor, renal epithelioid angiomyolipoma, metastasis

## Abstract

Malignant renal epithelioid angiomyolipoma (EAML) is an extremely rare disease with a poor prognosis, and currently there are no uniform criteria for its biological behavior. Here, we present a case of malignant renal EAML with TFE3 gene amplification in a 53-year-old woman. Four months after surgery, unenhanced computed tomography scans showed recurrence as well as metastasis in the abdomen and lung. The patient succumbed to rapid neoplastic progression of the disease 6 months later.

## Introduction 

Epithelioid angiomyolipoma (EAML) is a rare variant of angiomyolipoma with malignant potential and is an independent tumor subtype within the family of perivascular epithelioid cell tumors [1]. It has also been reported EAML sometimes exhibits aggressive growth, rupture, metastasis, or local recurrence [2, 3], posing a problem in differential diagnosis with renal cell carcinoma (RCC). Here, we report a case of malignant renal EAML with TFE3 gene amplification mimicking RCC. 

## Case report 

**Figure 1.**
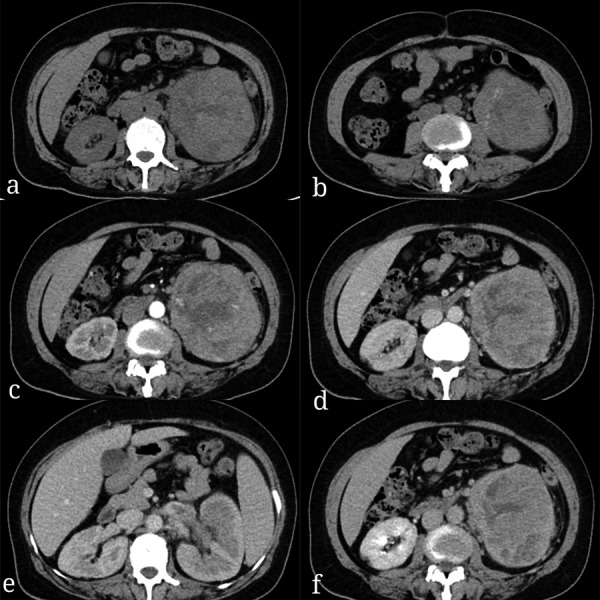
a, b: Precontrast computed tomography images showed a heterogeneous density mass with spot calcification. c: The mass showed moderate heterogeneous enhancement on corticomedullary phase with necrosis and many tumor vessels. d: The tumor showed continued enhancement on the nephrographic phase and (f) slight washout on the delayed phase. e: The filling defect in the left renal vein and enlarged retroperitoneal lymph nodes are shown.

A 53-year-old woman presented with a 5-day history of whole range painless, gross hematuria with presence of lumbago and fatigue over the past 6 months. Sonography showed a solid mass in the left kidney. Unenhanced computed tomography (CT) scans showed an ill-defined, irregular, slightly hyperdense mass (with size 11.9 × 10.0 × 10.1 cm) relative to renal parenchyma in the left kidney with corticomedullary involvement (Figure 1a). The mass had heterogeneous density with hypodense components in the center and spot calcifications at its low part (Figure 1b). The mass showed heterogeneous, moderate enhancement during the corticomedullary phase with necrosis in the center and many tumor vessels, continued enhancement during the nephrographic phase, and slight washout during the delayed phase (Figure 1c, d, f). The tumor was also found in the left renal vein (Figure 1e) and there were many enlarged retroperitoneal lymph nodes with a short-axis diameter of least 10 mm (Figure 1e). Radiographically, the mass was considered to be RCC. 

Laparoscopic radical nephrectomy with tumor thrombectomy and retroperitoneal lymphadenectomy were performed. At surgery, the kidney was obviously increased in size. The left renal vein was dilated and completely filled by thrombi. Upon histological examination, the specimen was grayish yellow, and necrosis and hemorrhage were observed. Tumor cells were cytologically malignant and exhibited marked pleomorphism as well as atypical mitotic figures (Figure 2a). Immunohistochemical staining confirmed that the epithelioid cells focally expressed HMB-45 and were positive for Melan-A and TFE3 (Figure 2b, c, d). Thus, the pathological diagnosis was EAML with malignant tendency. In the present case, fluorescence in situ hybridization (FISH) suggested X chromosome polyploidy resulting in TFE3 gene amplification without TFE3 gene fusion (Figure 2e). 

**Figure 3.**
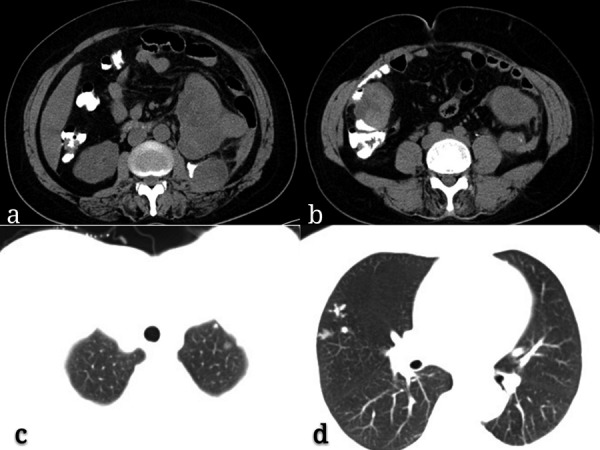
a, b: Unenhanced computed tomography images showed recrudescence and abdomen metastases. c, d: Multiple lung metastases are shown.

Four months after surgery, an unenhanced CT scan showed recrudescence as well as multiple abdomen and lung metastases (Figure 3a, b, c, d, e). The patient succumbed to neoplastic progression of the disease 6 months later. 

## Discussion 

EAML of the kidney is rare and strongly associated with tuberous sclerosis which was not observed in the present case characterized by the proliferation of predominant epithelioid cells [4]. Its malignant variant is uncommon, extremely aggressive [5], and behaves like RCC. Patient age ranges are from 14 to 68 years, with a mean age of 41 years without gender differences. Some EAMLs are related to the TFE3 gene, but the majority is related to TFE3 gene fusions. However, the case we presented was malignant renal EAML with TFE3 gene amplification. 

EAMLs typically present as large masses with intratumoral hemorrhage and necrosis, average 7 cm in size, and are typically much larger than fat-poor angiomyolipomas. They are hyperattenuating on unenhanced CT (≥ 45 HU) and T2-hypointense, which is attributed to their epithelioid muscle component [6, 7]. EAMLS may be heterogeneously, or less frequently homogeneously enhancing [8]. Malignant EAML may be difficult to diagnose due to the low abundance of adipocytes in the tumor. Thus, fat density does not appear to be crucial for distinguishing malignant EAML from angiomyolipoma. In the present case, CT scans identified a renal mass that exhibited central low attenuation consistent with necrosis, in addition to multiple enlarged retroperitoneal lymph nodes and a renal vein tumor. This CT appearance was similar to typical RCC [9], thus raising the suspicion of malignancy. Therefore, awareness of these radiological findings may facilitate the detection of malignant EAML. 

Currently, there are no uniform histological criteria for malignant EAML with the exception of distant metastases. Histologically, EAML is characterized by the predominant presence of polygonal epithelioid cells with atypical nuclei, mitotic figures, necrosis, marked atypical large cells with abundant eosinophilic cytoplasm that stain strongly for HMB-45, and low number of adipose cells. In the present case, FISH analysis suggested X chromosome polyploidy resulted in TFE3 gene amplification without TFE3 gene fusion. The differential diagnosis of malignant EAML includes clear cell RCC and Xp11.2/TFE3 RCC. Clear cell RCC typically shows significant heterogeneous enhancement and a significant washout. Stronger enhancement equivalent to the renal cortex is shown only in clear cell RCCs and not in other subtypes. Xp11.2/TFE3 RCC usually affects children more than adults, showing heterogeneity moderately prolonged the enhancement on dynamic contrast-enhanced CT. Circular calcification around or within the tumor is a specific clue for CT diagnosis of Xp11.2/TFE3 RCC. Currently, there is no known effective therapy for malignant EAML other than surgery [10]. However, nephrectomy alone may be inadequate in certain cases, and adjuvant therapy should be considered [2].Chemotherapy for malignant EAML is still under debate, although a number of patients with EAML have been reported to respond to doxorubicin [11]. 

The recognition of EAML is important, as it is either premalignant or malignant. In our case, the growth pattern of malignant renal EAML with TFE3 gene amplification was more aggressive and the prognosis was poorer compared with typical EAML. Thus, whenever a solid renal mass with necrotic and hemorrhagic portion and no fat component are encountered, even if accompanied by renal vein thrombosis and enlarged lymph nodes, the possibility of malignant EAML with TFE3 gene amplification should be considered. 

## Ethical approval 

We received institutional review board approval for this retrospective study. 

## Funding 

Shandong Research and Development Program (2016GSF201095). 

Research Award Fund for Outstanding Young-Middle aged Scientists of Shandong Province (BS2014YY004). 

## Conflict of interest 

None declared. 

**Figure 2.**
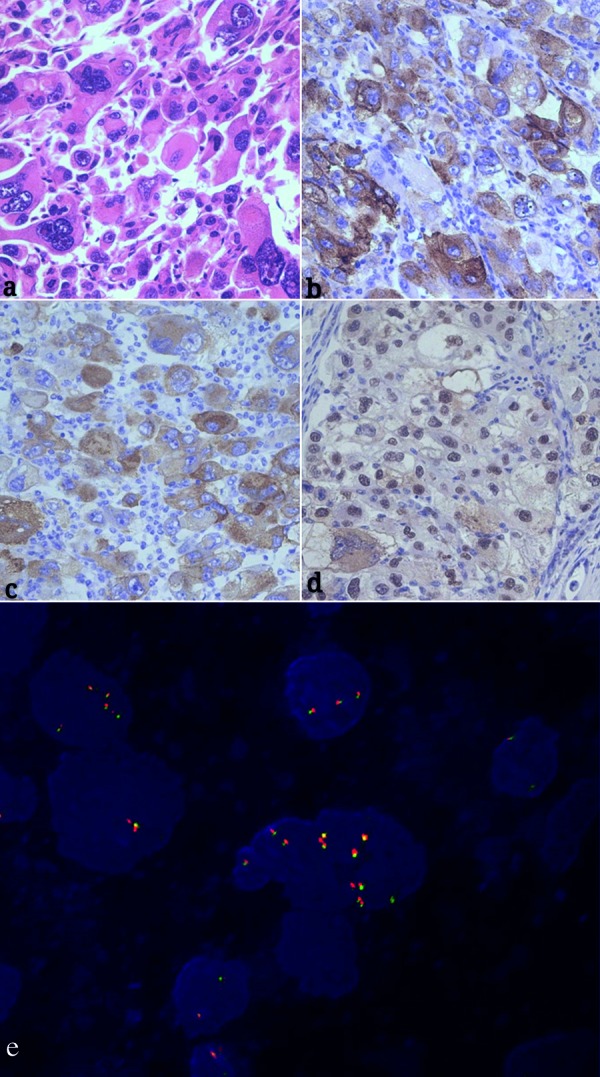
a – e: Hematoxylin and eosin staining and immunohistochemical staining (magnification × 400). a: Marked atypical large cells with abundant eosinophilic cytoplasm and atypical mitotic figures are shown. b, c, d: Tumor cells were focally expressed HMB-45 and positive for Melan-A and TFE3. e: Fluorescence in situ hybridization analysis (magnification × 1,000): TFE3 centromere is labeled as green fluorescence and TFE3 telomere is labeled as red fluorescence. There were many red and green fusion signals in the cell nucleus and no separation signal was found, suggesting X chromosome polyploidy resulting in TFE3 gene amplification.
